# Development of a Solvability Map

**DOI:** 10.18103/mra.v10i11.3312

**Published:** 2022-11-28

**Authors:** Gengsheng L. Zeng

**Affiliations:** 1 Department Computer Science, Utah Valley University, Orem, UT 84058, USA; 2 Department of Radiology and Imaging Sciences, University of Utah, Salt Lake City, UT 84108 USA

**Keywords:** Internal problem, Inverse problem, Image reconstruction, Biomedical imaging, Computed Tomography, Computer simulations, Monte Carlo

## Abstract

From time to time, it is necessary to determine whether there are sufficient measurements for the image reconstruction task especially when a non-standard scanning geometry is used. When the imaging system can be approximately modeled as a system of linear equations, the condition number of the system matrix indicates whether the entire system can be stably solved as a whole. When the system as a whole cannot be stably solved, the Moore-Penrose pseudo inverse matrix can be evaluated through the singular value decomposition (SVD) and then a generalized solution can be obtained. However, these methods are not practical because they require the computer memory to store the whole system matrix, which is often too large to store. Also, we do not know if the generalized solution is good enough for the application in mind. This paper proposes a practical image solvability map, which can be obtained for any practical image reconstruction algorithm. This image solvability map measures the reconstruction errors for each location using a large number of computer-simulated random phantoms. In other words, the map is generated by a Monte Carlo approach.

## I. INTRODUCTION

Data sufficiency conditions for continuous measurements were developed for many imaging geometries. For example, Orlov’s condition uses the great-circle criterion to determine whether a positron emission tomography (PET) system measures a complete data set for analytical three-dimensional (3D) image reconstruction^1^. In Orlov’s condition, the PET detector size is assumed to be infinity, and the sampling is assumed to be continuous. If the normal direction trajectory of the PET detector contains a great circle, the data set is sufficient. Orlov’s condition considers the 3D parallel line integral measurements. Tuy’s condition, on the other hand, considers the 3D cone-beam line integral measurements^2^. Tuy’s condition is able to verify if a 3D cone-beam imaging system acquires a complete data set. Tuy’s condition states that if every plane that cuts through the object intersects the cone-beam focal-point trajectory, the data set is sufficient for the reconstruction of the object. Once again, the detector is assumed to be infinite, and the sampling is continuous. A more general data sufficiency condition in the n-dimensional complex space is proposed by Kirokov^4^.

For discrete sampling, the detector takes discrete finite number of positions, and the detector consists of discrete finite number of detection cells. The detector size is finite, which may lead to data truncation, where the detector does not cover the entire object. The common practice in processing discrete measurements is to use a linear model, which formulates the imaging process as a system of linear equations *AX = P.* The unknowns (i.e., the variables), *X*, of the system are the image pixels or voxels. The coefficient matrix (also known as the system matrix), *A,* is assumed to be known. The measurements, *P*, are the constant terms. The condition number analysis is a classic approach to investigate whether the normal equations *A*^*T*^*AX=A*^*T*^*P* is stably solvable^5^. Singular value decomposition (SVD) analysis is able to diagnose invertibility and noise sensitivity of systems of linear equations. In reference 5, the condition number (i.e., the ratio of maximal and minimal singular values of matrix *A)* was calculated using the Lanczos iterative method^6^ for image volumes of 65 × 65 × 128. Some cone-beam imaging trajectories were analyzed and compared using the condition number analysis; a circular sine-wave trajectory was determined to be the most stable sampling scheme among the orbits investigated^5^. One drawback of the condition number analysis is that it does not work in region-of-interest (ROI) reconstruction with truncated data, because the system of equations is under-determined, and the associated condition number is essentially infinity. In this case the inverse matrix of *A*^*T*^*A* does not exist.

In situations where *A*^*T*^*A* is singular, the Moore-Penrose pseudo inverse matrix, *A*^*+*^, can help^6^. If *A* is an *n* × *m* matrix, then *A*^*+*^ is an *m* × *n* matrix. In general, *A*^*+*^*A ≠ I*, where *I* is the *m* × *m* identity matrix. A method in Reference 6 was proposed to identify the solvable subset of the unknowns. The method in Reference 6 used the diagonal elements of *A*^*+*^*A* as a map. Each diagonal element of *A*^*+*^*A* corresponded to an image pixel. If a diagonal element is one, the corresponding image pixel can be reconstructed. A drawback of this method is that the Moore-Penrose pseudo inverse matrix *A*^*+*^ is not easy to compute for a large imaging system, because the singular value decomposition (SVD) is required to perform on a large matrix^7^, which requires a huge amount of computer memory.

This paper proposes a method to overcome the drawback in Reference 6 so that the SVD computation is not required. This new method is Monte Carlo based and is described in Section 2 of this paper. The computer simulation results are presented in Section 3.

## II. METHODS

### A. Region-of-interest (ROI) image reconstruction

One of the following situations can happen when an object is not completely measured. The first situation is due to the limited detector size, and only a portion of the object can be seen by the detector. The second situation is due to the lack of angular coverage. When the measurements are insufficient, it is likely that we are unable to have a stable reconstruction of the entire object. However, we may be able to have a stable reconstruction of a subset of the knowns. The aim of this paper is to determine such a subset if it exists.

### B. Proposed method

Let us consider a generic image reconstruction algorithm, G; it can be an iterative or non-iterative algorithm; it can be a linear or nonlinear algorithm. For example, this generic image reconstruction algorithm, G, can be the iterative gradient descent (GD) algorithm, or a variate of the GD algorithm tailored for the data truncation, or a maximum-likelihood expectation-maximization (MLEM) algorithm, and so on.

We use computer simulation to create a large number of random objects, generate their projection measurements, add noise to the measurements, reconstruct the images, and compute the error between the reconstructed images and the true images. Finally, calculate the average error image for these large number of random objects. This average error image is our proposed image solvability map.

### C. Avoiding the inverse crime

When a physical continuous system is modeled as a discrete system, modelling errors exist^8^. It is an inverse problem crime when these errors are ignored in developing and analyzing an inverse solution. For example, a typical inverse crime during computer simulations is to use the same generator to create the measurements and to be used in the reconstruction algorithm. To avoid inverse crime, in our case, if *G* is used to creating computer simulated measurements, *G* is not allowed to be used as the forward projection operator in the reconstruction algorithm.

In the computer simulations in this paper, the measurement generation uses random phantoms with the size of 384 × 384, and after projections are computed, the three adjacent projection bins are combined. Some Poisson noise is then incorporated in the combined measurements. In the image reconstruction, the image size is 128 × 128.

### D. Imaging geometry

**FIGURE 1.**
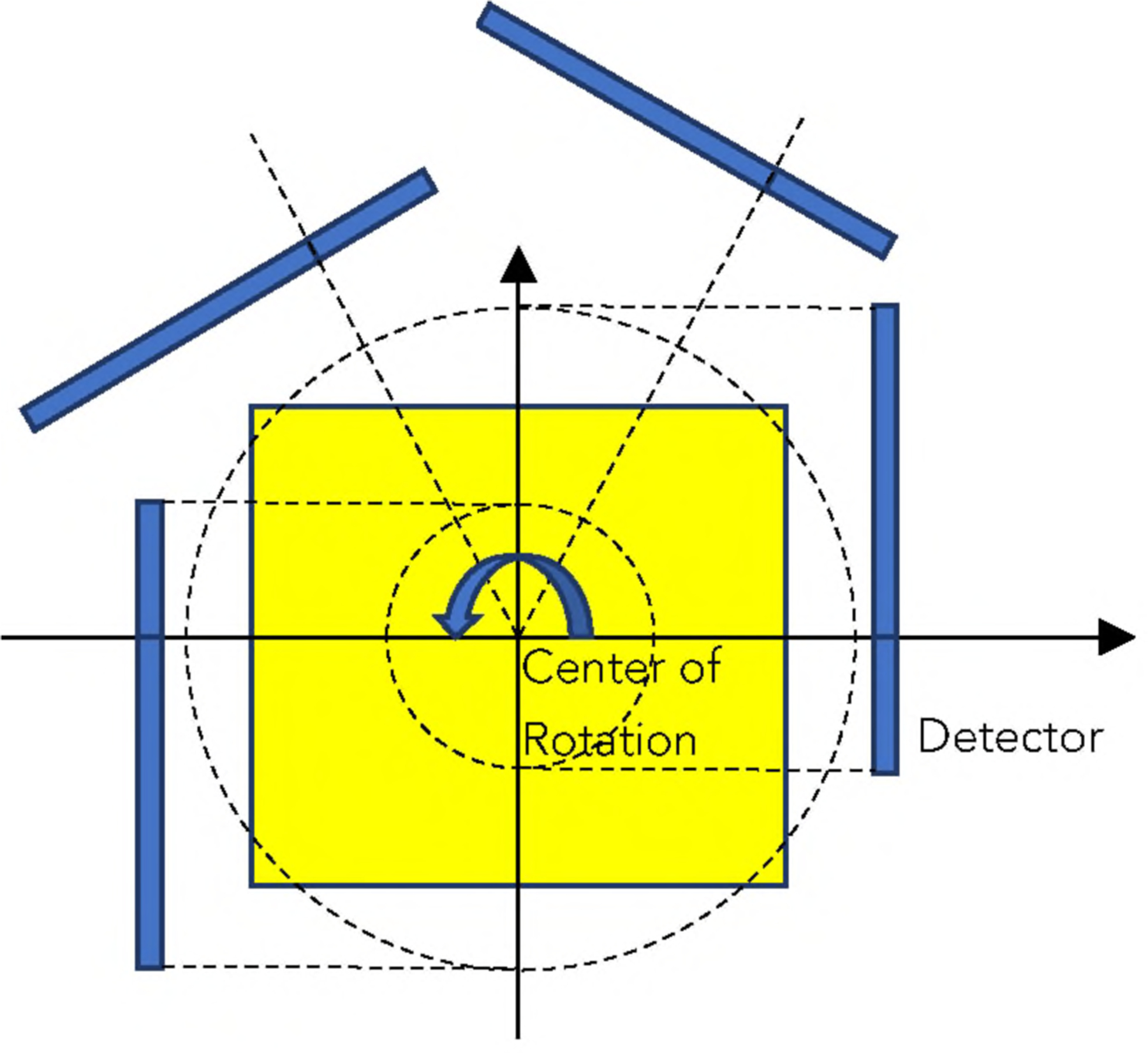
A hypothetical parallel-beam imaging system.

A hypothetical parallel-beam imaging system was simulated. The detector was asymmetric about the axis of rotation as shown in Fig. 1. The detector rotated 180° with 180 stops. In other words, the angular interval was 1°.

**FIGURE 2.**
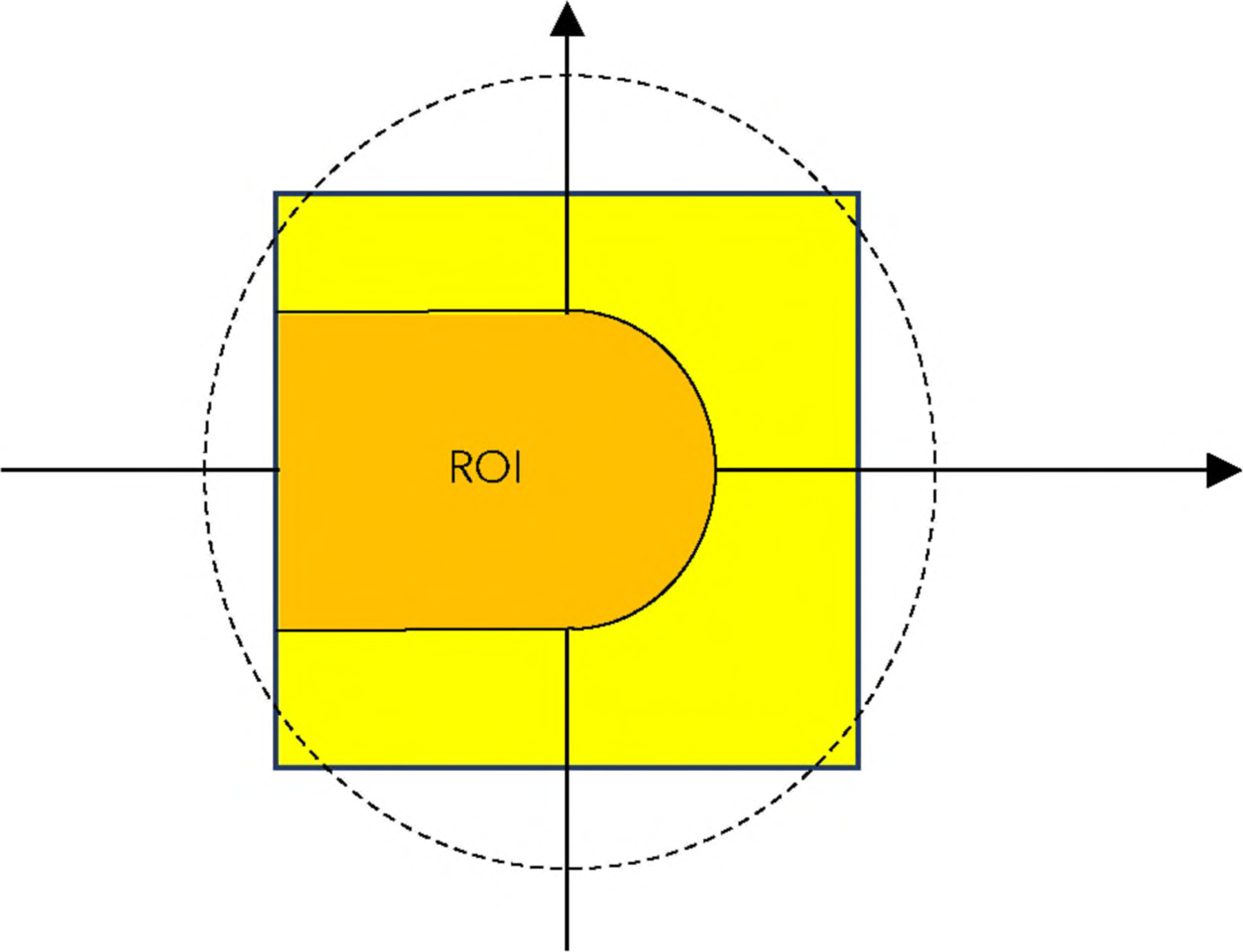
The points in the ROI have 180° angular measurements.

In 2D tomography, according to Kirokov’s criterion^3^, a point is fully measured if all lines passing through that point are measured. In Fig. 2, the ROI indicates the region of points that are fully measured. If an object is completely contained in the ROI, the object can be stably reconstructed, under the conditions that the projections are not truncated, and the number of views is sufficient.

### E. Image reconstruction algorithms

In this paper, the iterative gradient descent (GD) algorithm and the iterative maximum-likelihood expectation-maximization (ML-EM) algorithm are considered to test the feasibility of the proposed method^9^. These two algorithms are well-known in medical imaging community. When the projection data set is not complete or truncated, these two original algorithms may not work well. These two algorithms have many variations. The GD algorithm and the ML-EM algorithm are expressed in (1) and (2), respectively^9^.

(1) $$GD:x_{i}^{\left(k+1\right)}=x_{i}^{\left(k\right)}-\alpha \sum_{j}a_{ij}\left(\sum_{n}a_{nj}x_{n}^{\left(k\right)}-p_{j}\right)$$

(2) $$ML-EM:x_{i}^{\left(k+1\right)}=\frac{x_{i}^{\left(k\right)}}{\sum_{j}a_{ij}}\sum_{j}a_{ij}\frac{p_{j}}{\sum_{n}a_{nj}x_{n}^{\left(k\right)}}$$

where

$x_{i}^{\left(k\right)}$ is an element in image *X* and is the *i*th image pixel value at the *k*th iteration;

*a*_*ij*_ is an element in the system matrix *A* and is the contribution from the *i*th image pixel to the *j*th projection bin;

*P*_*j*_ is an element in projections *P* and is the *j*th projection value;

*k* is the iteration number;

*α* is the step size for the GD algorithm.

**FIGURE 3.**
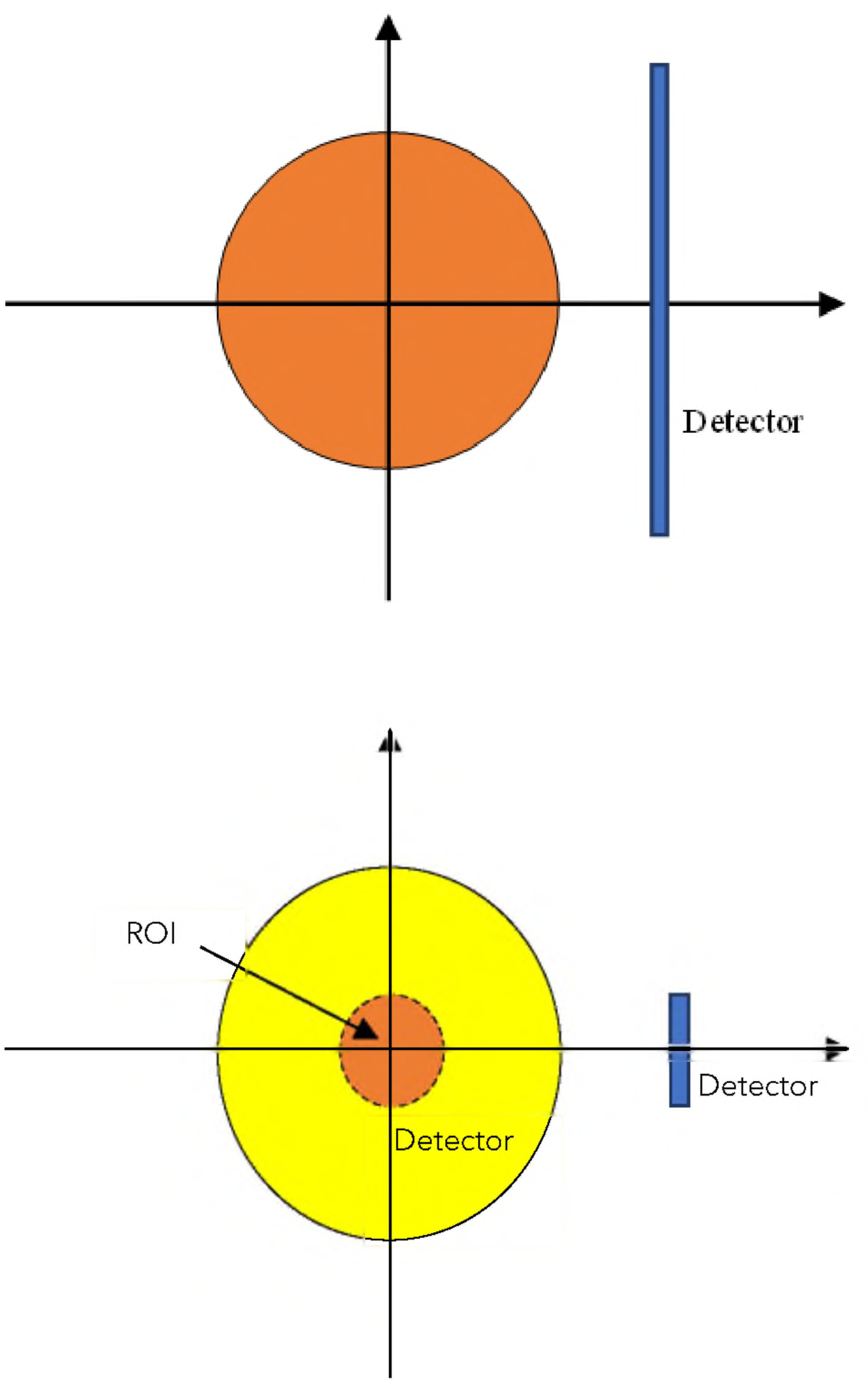
Upper: Every point in the object is fully measured when the detector rotates 180°. Lower: An internal problem is shown where the detector is too small to cover the entire object and truncation happens at both sides of the detector.

When the object is larger than the detector and the projections are truncated at both ends of the detector, the image reconstruction problem is referred to as the internal problem^10^. It is known that the internal problem is unsolvable ^10^. A support of an object is an image, whose pixel value is non-zero (say, value one) if the corresponding object value is non-zero at the same location. If the support of the object is known, using the support information can improve the reconstruction in an internal problem^11^. An internal problem is illustrated in right part of Fig. 3.

For the GD algorithm, we enforce the finite support at every iteration as

(3) $$x_{i}^{\left(k+1\right)}=0\text{ if pixel }x_{i}\text{ is not in the support.}$$

For the ML-EM algorithm, we only need to enforce the finite support at the initial condition as

(4) $$x_{i}^{\left(0\right)}=0\text{ if pixel }x_{i}\text{ is not in the support,}$$

because a pixel is zero at any iteration, the pixel will remain zero thereafter in the ML-EM algorithm. It is recommended that whenever using truncated projections in an iterative algorithm, the image array be large enough to contain the entire object even though the detector is not large enough to see the entire object^11^.

For the truncated data, a simple *modified* method can be used to reduce the artifacts^12^. This modified method assumes that if *p*_*j*_ is not measured, *p*_*j*_ is assigned to the forward projection value $\sum_{n}a_{nj}x_{n}^{\left(k\right)}$ at each iteration *k* for both the GD and the ML-EM algorithms, that is,

(5) $$p_{j}=\sum_{n}a_{nj}x_{n}^{\left(k\right)},\text{ if }p_{j}\text{ is not measured}.$$

### F. Computer simulations

In this paper, each of the computer-generated phantoms consisted of four ellipses with random sizes, locations, and intensities. The phantom size was 384 × 384. A narrow Gaussian lowpass filter with a standard deviation of one was applied to smooth out the sharp edges a little. Next, the image was normalized to the range of [0, 1].

Line integrals were calculated using the parallel-beam imaging geometry shown in Fig. 1, where the detector was asymmetric, and the number of views was 180 over 180°. After the line integrals were calculated, Poisson noise was incorporated into the simulated line-integrals. Then, the three adjacent detector bins were combined into one detector bin. In other words, the new detector’s bin-size was three times larger than the original detector’s bin-size. The binned-down measurements were ready for image reconstruction into an image array with the size of 128 × 128.

There were two sets of simulated measurements. The first set consisted of 1000 random phantoms and was described in the paragraphs above. The second set contained the same 1000 random phantoms as in the first set; the only thing different from the first set was that the detector was large enough to see the entire phantom as indicated in left diagram in left part of Fig. 3. The detector in the first set was asymmetric and had 107 detection bins. The detector in the second set was symmetric and had 185 detection bins. The detector bin size was the same as the image pixel size.

The following six algorithms were used to reconstruct the images and were compared:

- Iterative gradient descent (GD) algorithm (1);
- Iterative GD algorithm with the finite support enforcement (3);
- Iterative GD algorithm with the truncation modification enforcement (5);
- Iterative GD algorithm with the finite support (3) and truncation modification (5) enforcements;
- Iterative maximum-likelihood expectation-maximization (ML-EM) algorithm (2);
- Iterative ML-EM algorithm with the finite support enforcement (4);
- Iterative ML-EM algorithm with the truncation modification enforcement (5);
- Iterative ML-EM algorithm with the finite support (4) and truncation modification (5) enforcements.

### G. Image solvability map

For each reconstructed image *X, a* squared-error image *E*(*X*) is calculated as

(6) $$e_{i}={\left(x_{i}-x_{i}^{true}\right)}^{2}$$

where *e*_*t*_ is the *i*th pixel in the squared-error image *E*(*X*), $x_{i}^{true}$ is the *i*th pixel in the true image *X*^*true*^, and *x*_*i*_ is the *i*th pixel in the reconstructed image *X*.

If *n* is the total number of random phantoms in the computer simulation (we had *n* = 1000 in this paper), the *image solvability map* is the average image of the squared-error images, that is

(7) $$\text{Image Solvability Map}=\frac{1}{n}\sum_{m=1}^{n}E\left(\text{the }m\text{th phantom's reconstruction}\right).$$

All image values in the image solvability map are non-negative. A smaller value in the map indicates that the corresponding pixel is more solvable. Due to the random noise and the determinist discrepancies introduced to fight the inverse problem crime, the minimum value in the image solvability map is not zero.

In order to visualize the map details when the pixel values are close to zero, the display values of the image solvability maps use the following non-linear transformations

(8) $$\nu_{0}=1-e^{-20\times v_{i}},$$

where *v*_*i*_, is the input value calculated from (7) and *v*_*o*_ is the output value to be displayed in figures. This non-linear transformation (8) translates [0, ∞) to [0,1). The large values in the map (7) are suppressed to close to one when they are displayed.

## III. RESULTS

**FIGURE 5.**
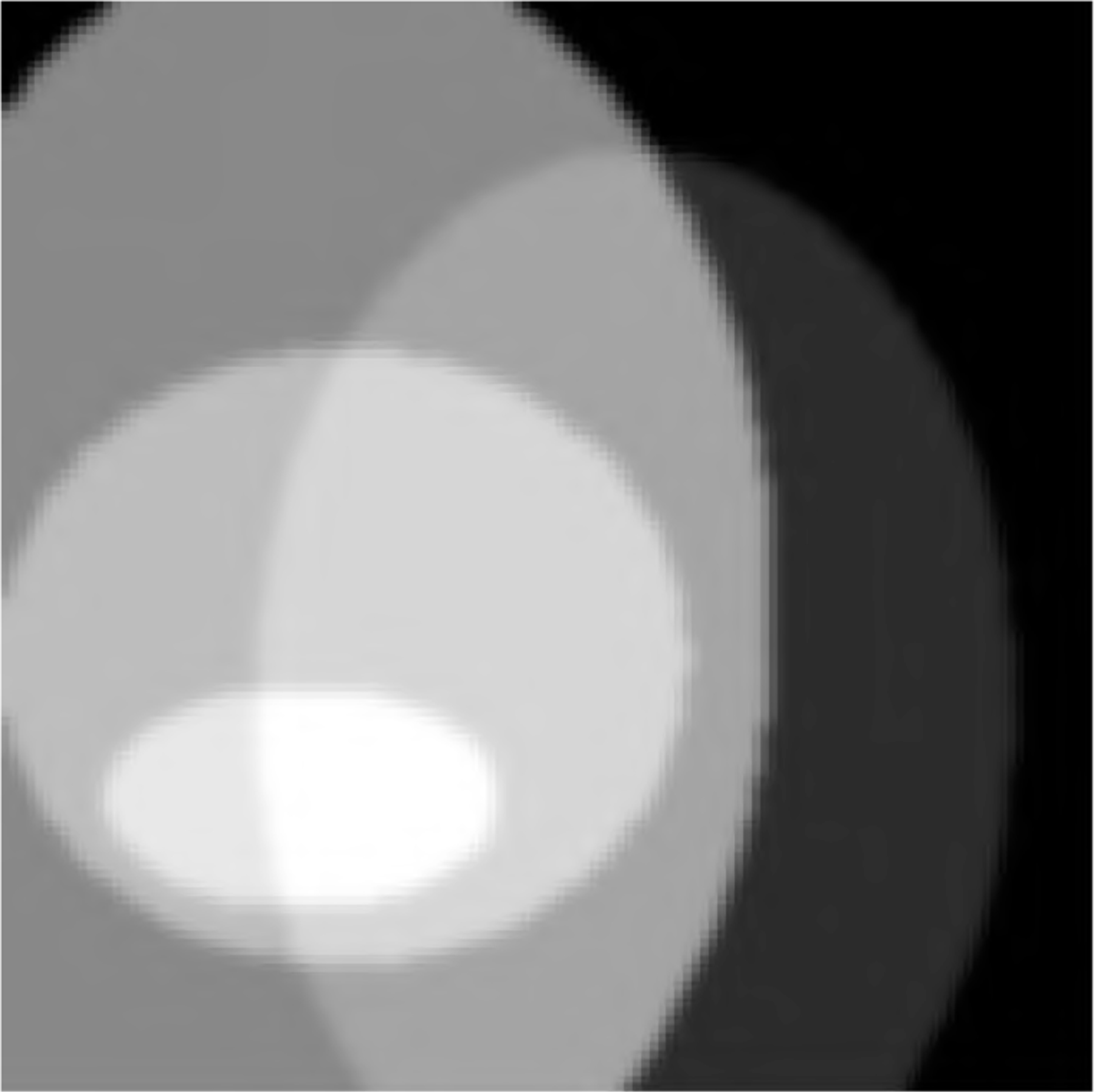
One of the 1000 random phantoms used in the computer simulations.

**FIGURE 6.**
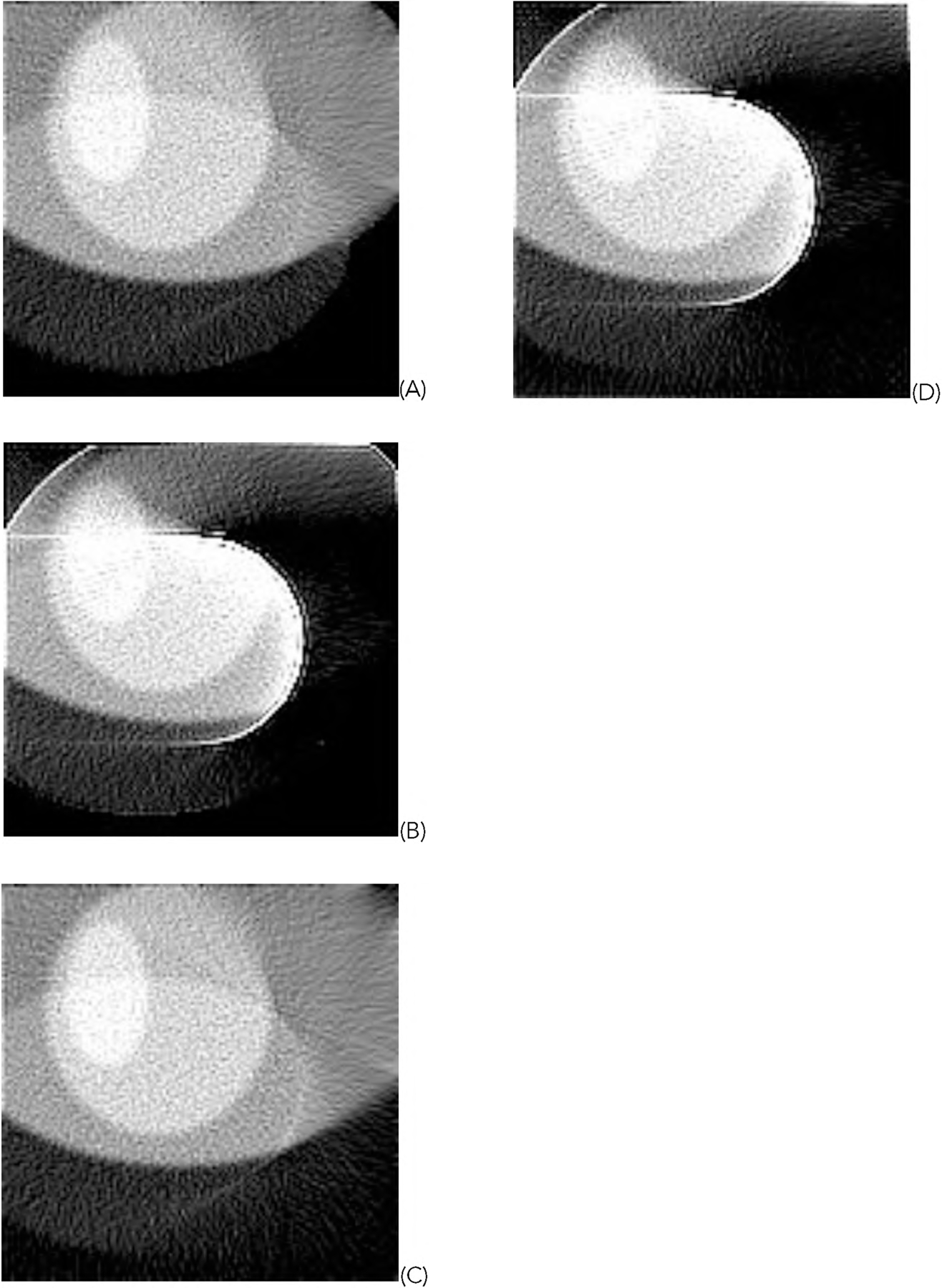
The images reconstructed with the GD algorithms using truncated data. (A) With formulas (1), (3), and (5); (B) With formulas (1) and (3); (C) With formulas (1) and (5); (D) With formula (1).

**FIGURE 7.**
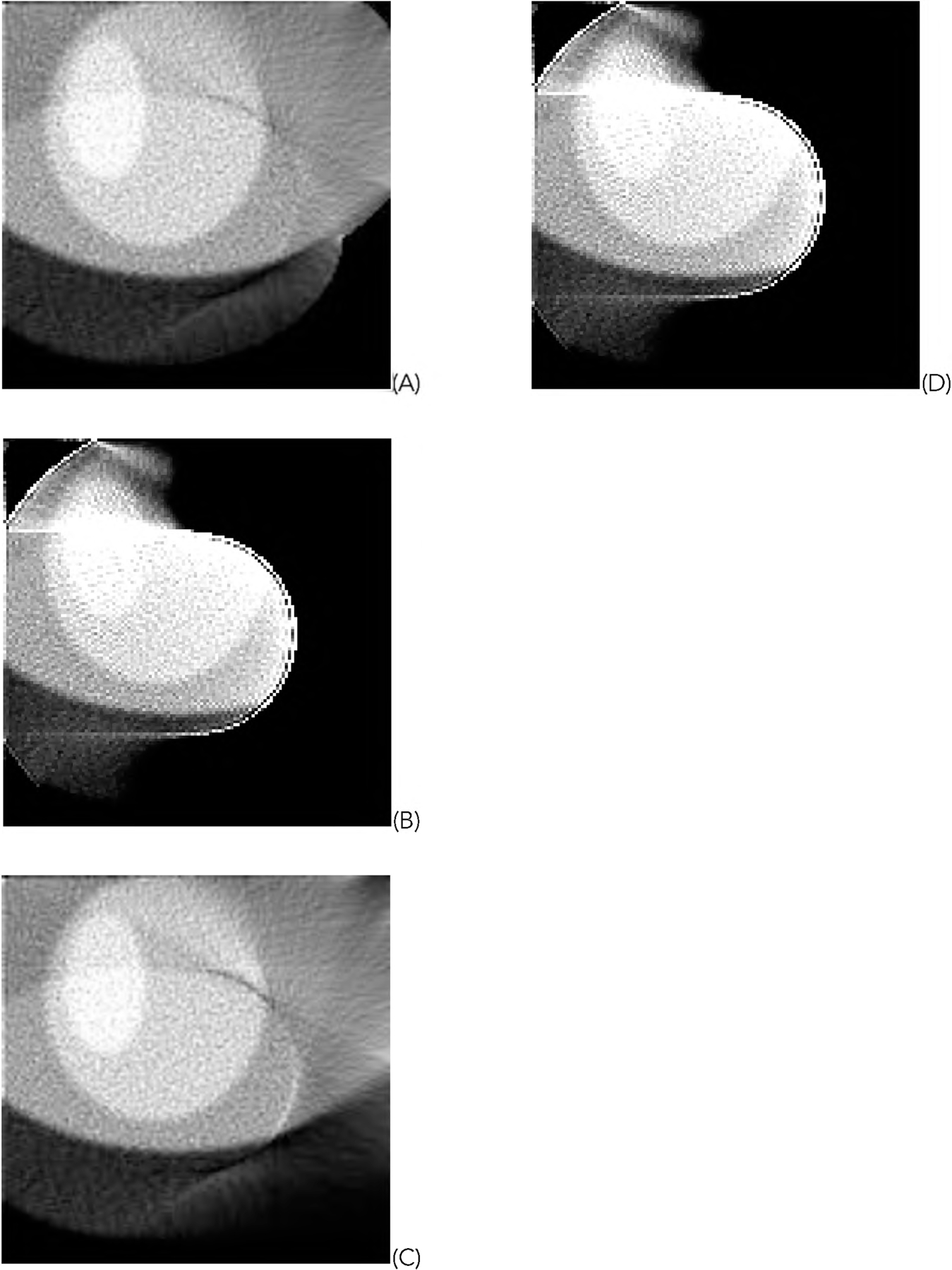
The images reconstructed with the ML-EM algorithms using truncated data. (A) With formulas (2), (4), and (5); (B) With formulas (2) and (4); (C) With formulas (2) and (5); (D) With formula (2).

Fig. 5 shows one representative of the 1000 random phantoms. The image reconstruction results from the first data set using truncated data are shown in Figs. 6 and 7 for the representative phantom shown in Fig. 5. The reconstruction algorithms are listed in the Part F of Section 2. The images in Fig. 6 are obtained from the gradient descent (GD) algorithms. The images in Fig. 7 are obtained from the ML-EM algorithms.

**FIGURE 8.**
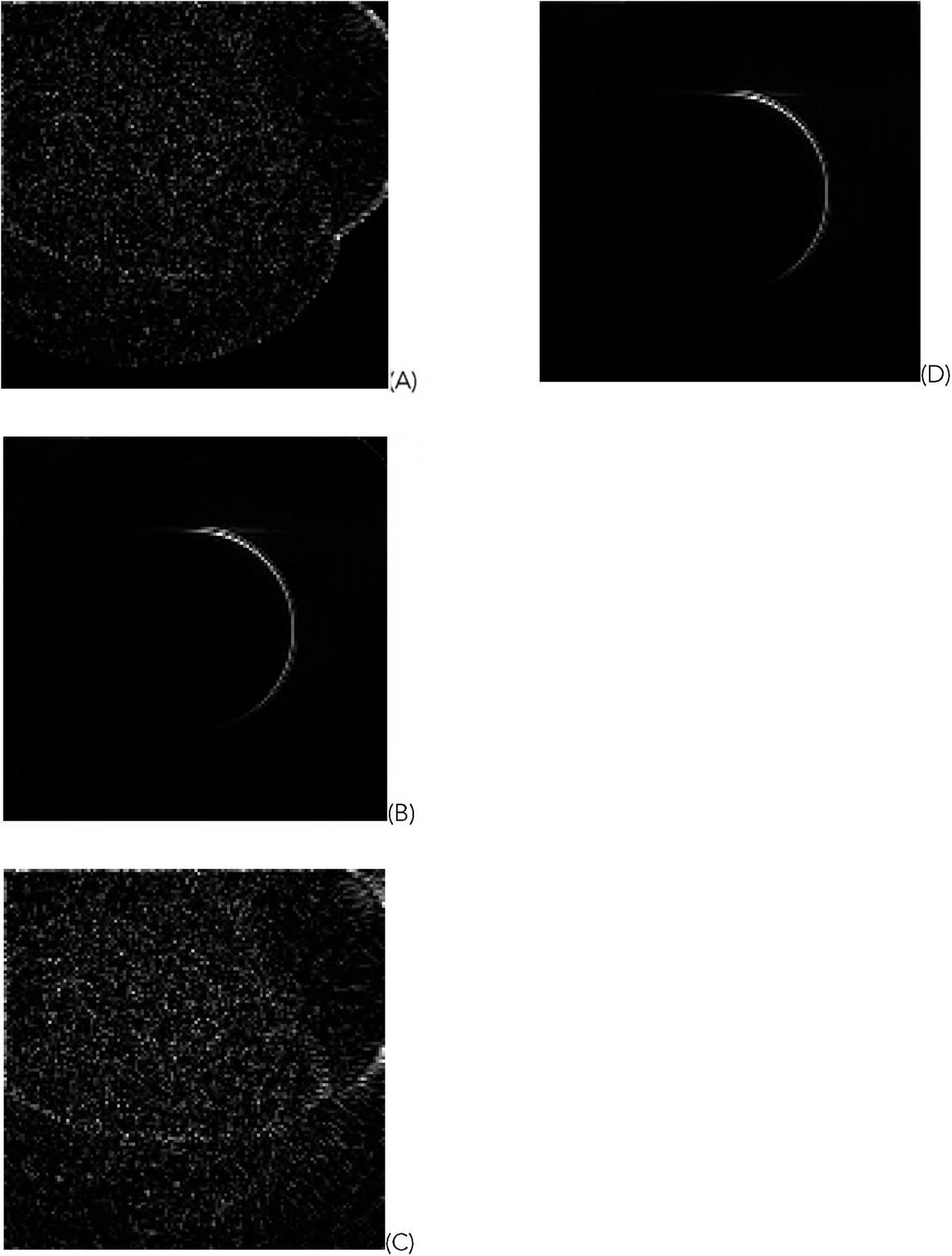
The squared-error images for the reconstructions with the GD algorithms using truncated data. (A) With formulas (1), (3), and (5); (B) With formulas (1) and (3); (C) With formulas (1) and (5); (D) With formula (1).

**FIGURE 9.**
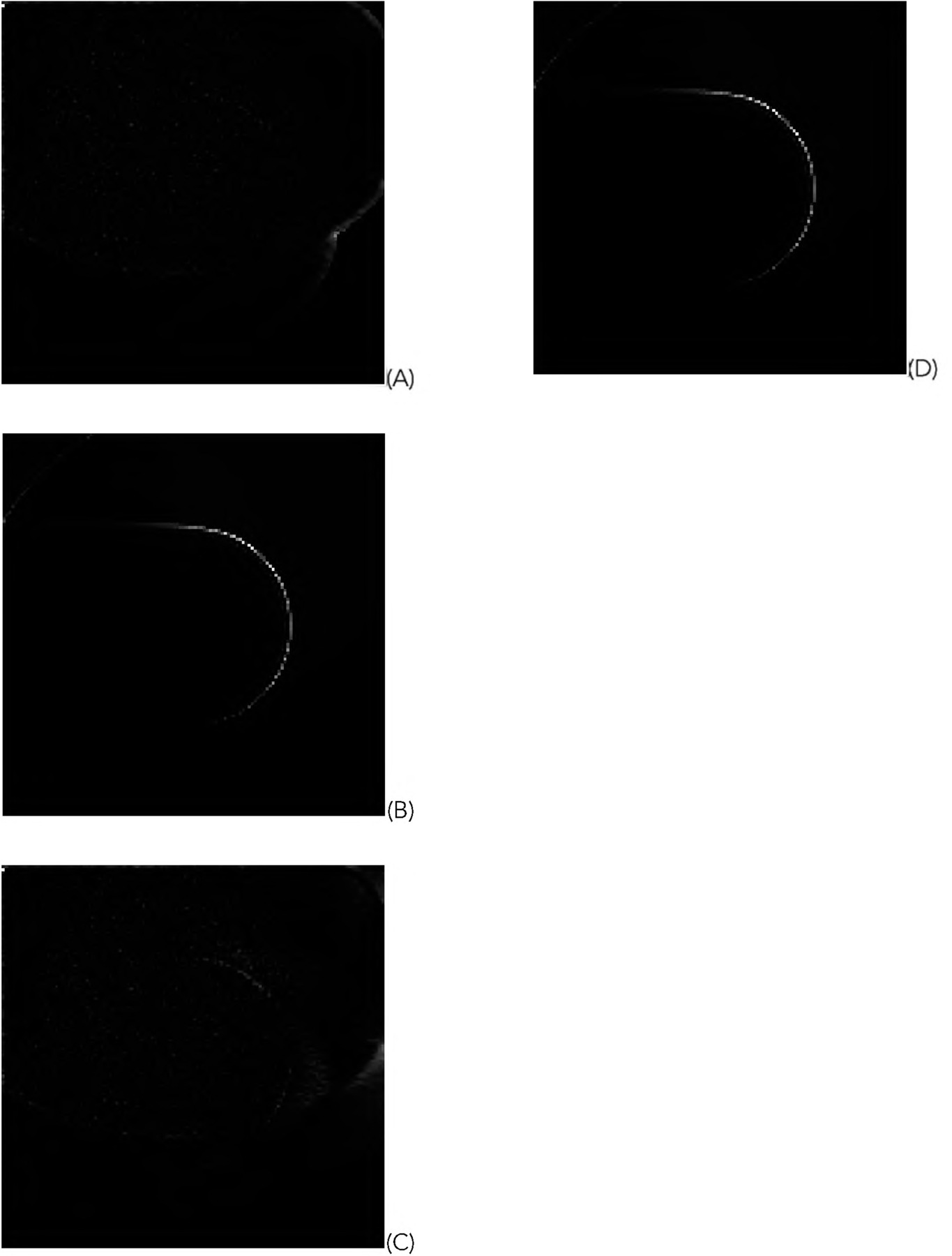
The squared-error images for the reconstructions with the ML-EM algorithms using truncated data. (A) With formulas (2), (4), and (5); (B) With formulas (2) and (4); (C) With formulas (2) and (5); (D) With formula (2).

**FIGURE 10.**
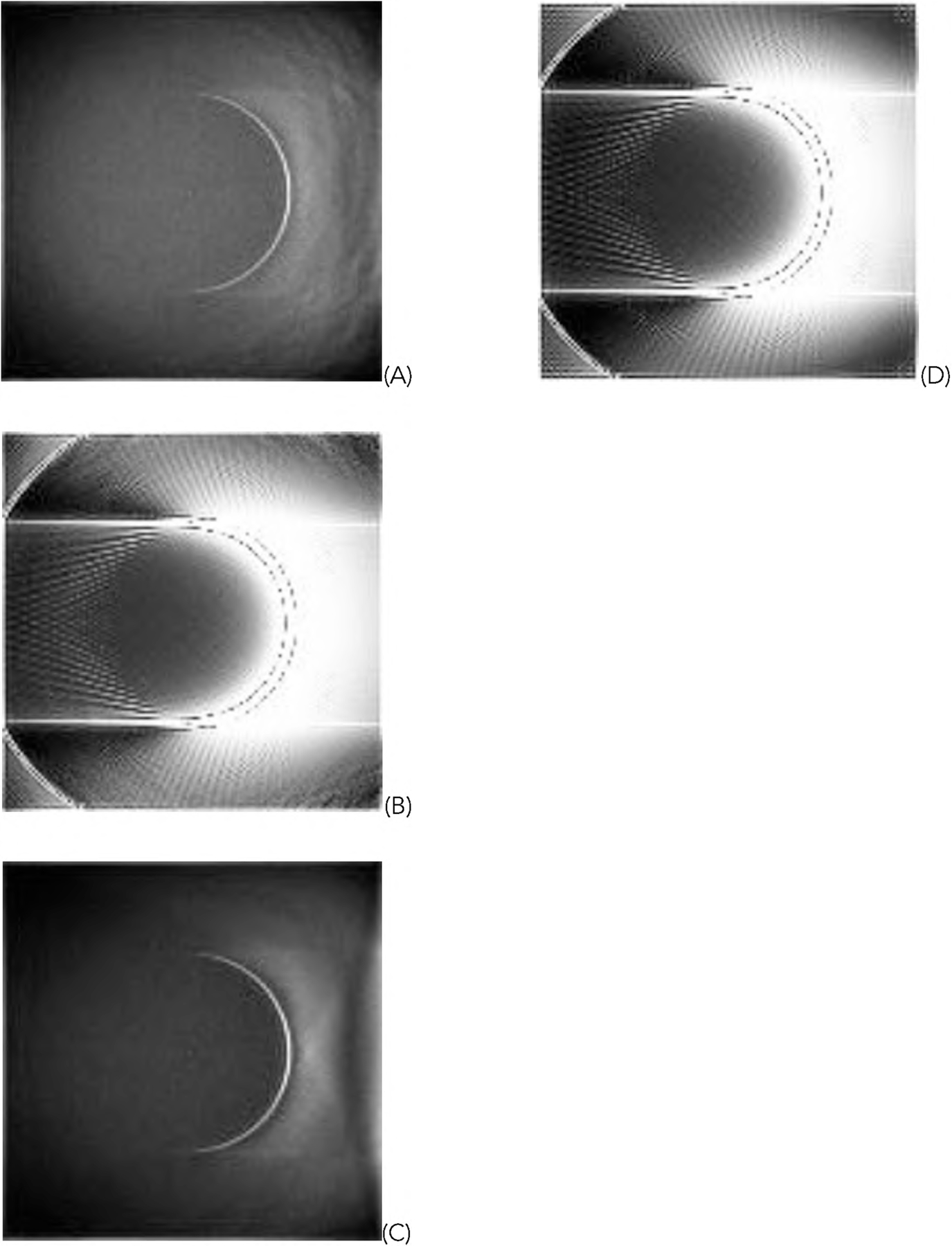
The image solvability maps for the reconstructions with the GD algorithms using truncated data. (A) With formulas (1), (3), and (5); (B) With formulas (1) and (3); (C) With formulas (1) and (5); (D) With formula (1).

**FIGURE 11.**
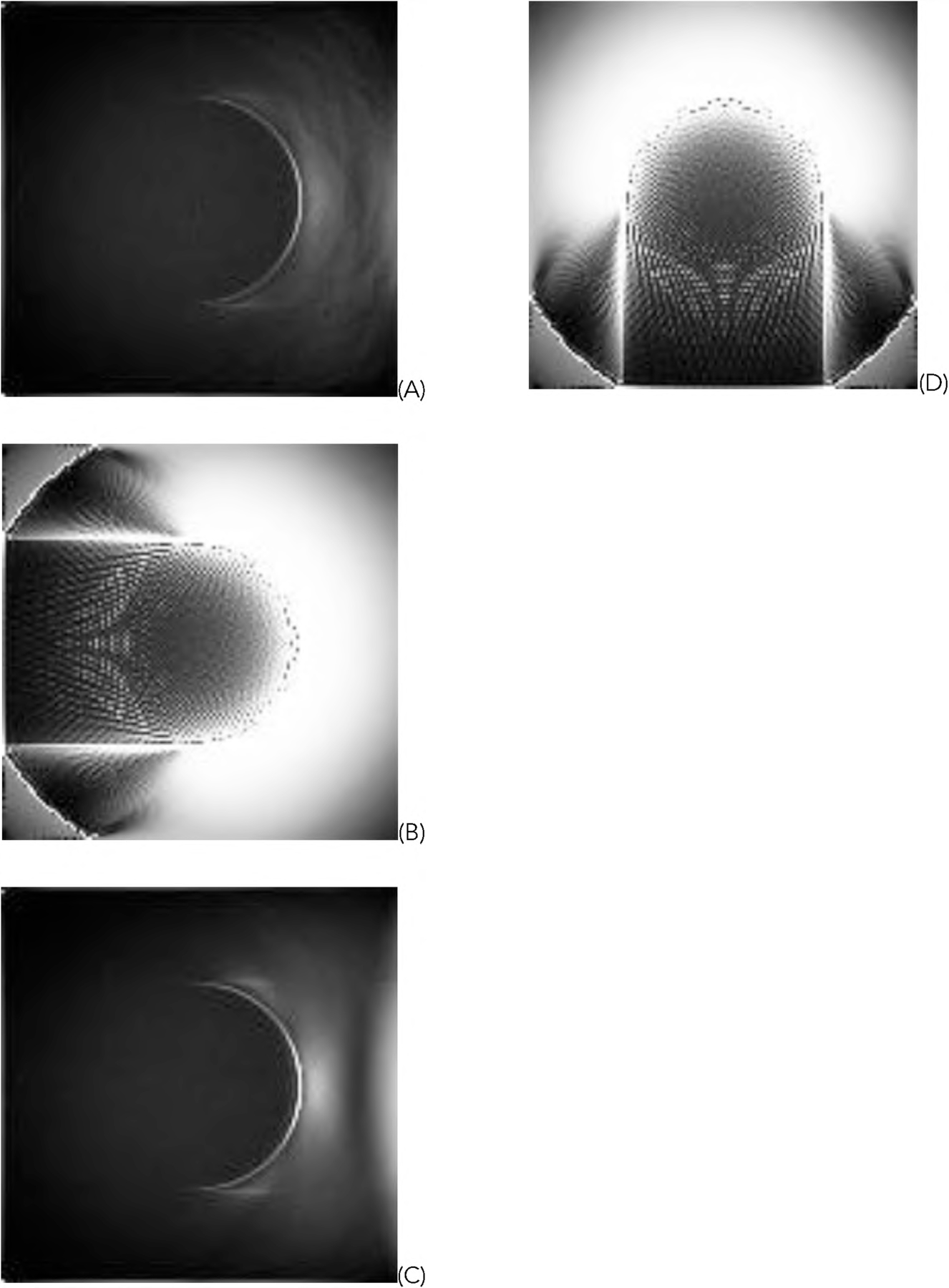
The image solvability maps for the reconstructions with the ML-EM algorithms using truncated data. (A) With formulas (2), (4), and (5); (B) With formulas (2) and (4); (C) With formulas (2) and (5); (D) With formula (2).

For the representative random phantom shown in Fig. 5, the squared-error images associated with the reconstructed images are shown in Figs. (8) and (9) for the GD and ML-EM algorithms, respectively. After finding the average of the 1000 squared-error images, an image solvability map is obtained. The image solvability maps for the 8 reconstruction algorithms are shown in Figs. 10 and 11, respectively.

**FIGURE 12.**
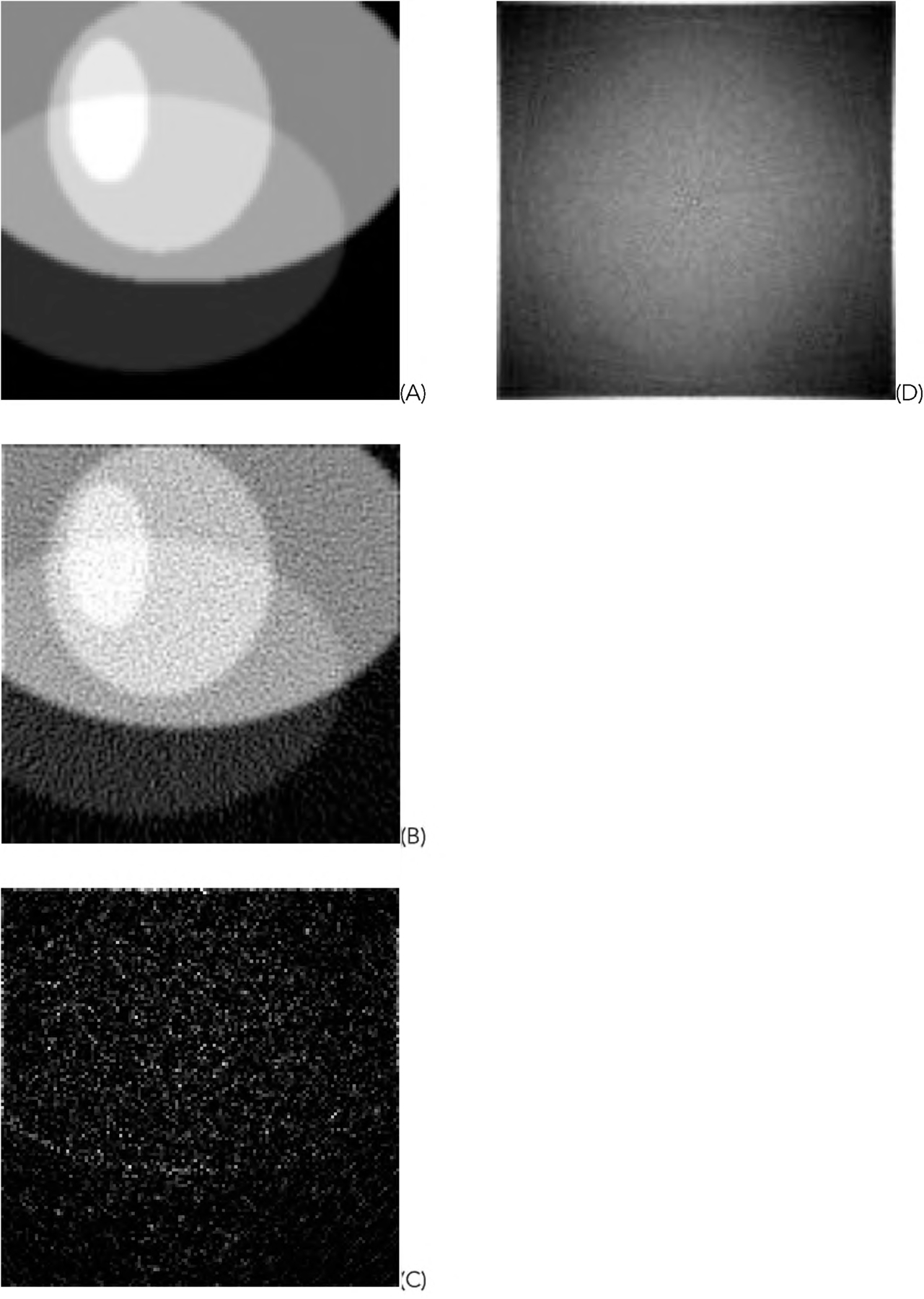
Results of the GD algorithm using the untruncated data. (A) The true phantom; (B) The reconstruction; (C) The squared-error image; (D) The image solvability map.

**FIGURE 13.**
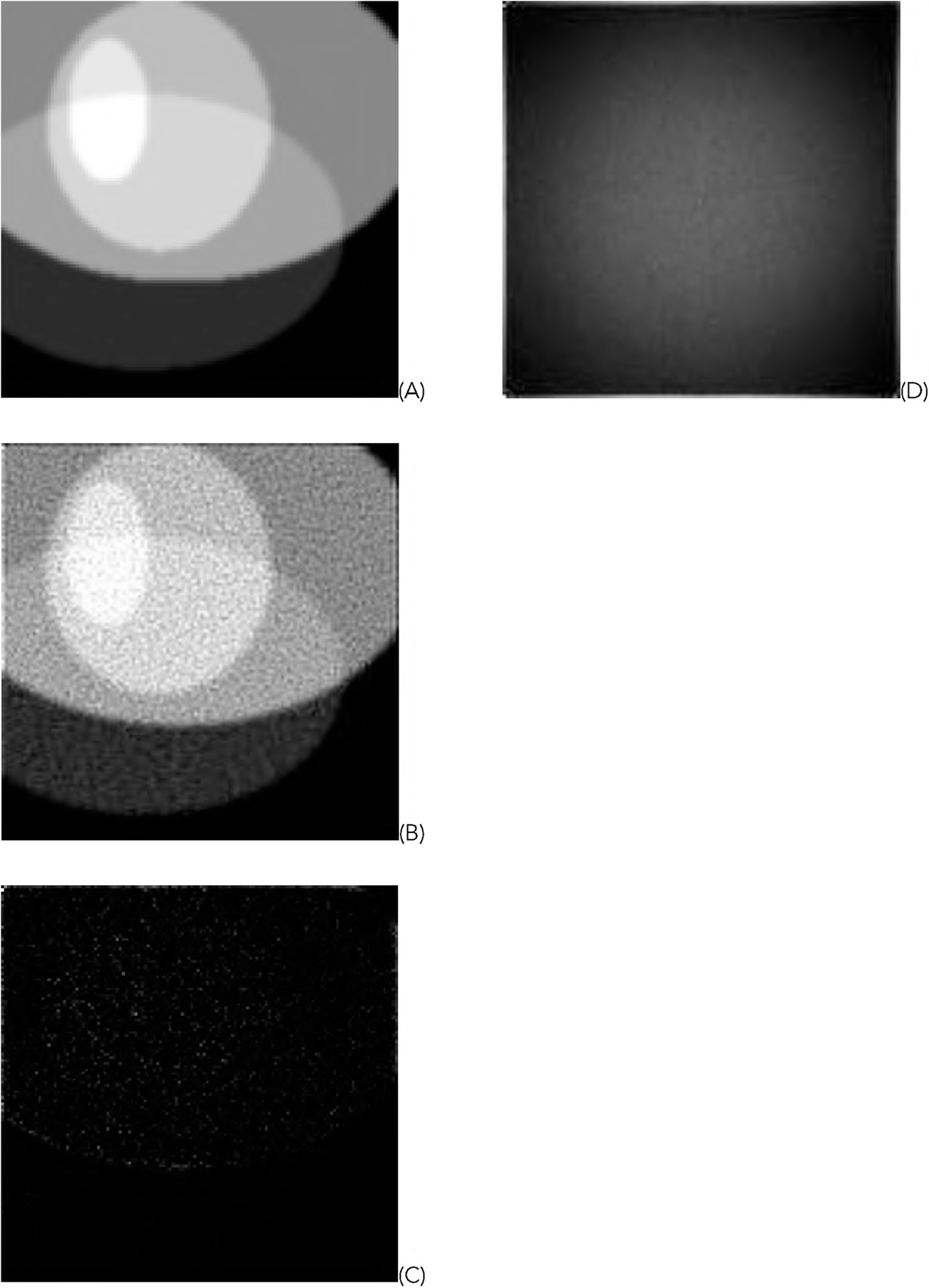
Results of the ML-EM algorithm using the untruncated data. (A) The true phantom; (B) The reconstruction; (C) The squared-error image; (D) The image solvability map.

In this paper, all phantom images (Fig. 5, (A) and (B) of Figs. 6, 7, 12, and 13) are displayed in the linear grayscale window of [0, 1], All squared-error images and image solvability maps are displayed from zero to the maximum pixel value in the image. The image solvability maps are displayed with a non-linear transformation (8) to emphasize the small values. The minimum and maximum values for the image solvability maps are listed in Tables 1 and 2.

For the comparison purposes, the results of using untruncated measurement data (i.e., the second data set) are shown in Figs. 12 and 13, for the GD algorithm and ML-EM algorithm, respectively. The minimum and maximum values for the image solvability maps are listed in Table 3.

## IV. DISCUSSION

From the results, we observe that the untruncated data gives much stabler reconstructions than the truncated data. Table 3 indicates that the image solvability maps for untruncated data are close to zero and the reconstruction are overall stable. Figures 12 and 13 show that the image solvability maps for untruncated data are smooth.

When the measurements are truncated as in the first data set, different algorithms have different performances. The image solvability maps in Figs. 10 and 11 have some dramatic differences for different regions. Whenever the truncation modification (5) is not used, the reconstruction looks very poor. In fact, the default naive implementation of (1) and (2) treats unmeasured projections as zeros; this naive implementation is not correct. The unmeasured projections should never be treated as zeros. The unmeasured projections should be discarded and not be included in the reconstructions (1) and (2). Formula (5) is an alternative way to discard the unmeasured projections. The image solvability maps thus can indicate which algorithm implementation is preferred and which regions the image can reconstructed stably.

The proposed solvability map is an extension of the data sufficiency conditions^1–3^, which assume continuous sampling. Currently, no data acquisition systems are able to acquire data continuously. On the other hand, the proposed method is based on discrete sampling and can be directly apply to a current state-of-the-art tomographic system.

The traditional data sufficiency conditions have binary outcomes^1–3^: satisfied or unsatisfied. When the conditions are satisfied, the whole object is able to be reconstructed. When the conditions are not satisfied, we do not know whether a sub region of the object can be reconstructed. On the other hand, our proposed method is a gray scale solvability image, which clearly displays which regions are more solvable than other rations.

Unlike the traditional data sufficiency conditions^1–3^, the proposed solvability map is reconstruction algorithm dependent. It can be used to compare image reconstruction algorithms and to evaluate the accuracy of the reconstruction algorithms.

One important application of the proposed image solvability map is in C-arm cone-beam imaging trajectory design^13–14^. Another important application is in region-of-interest (ROI) imaging system design^15–16^.

In fact, the proposed method has a broader impact on imaging systems, including transmission tomography, emission tomography, and magnetic resonance imaging (MRI). The applications of the proposed solvability map are well beyond the scope of truncation data effects. The map can evaluate the effectiveness of various data acquisition strategies and sampling strategies.

## V. CONCLUSIONS

It has been a desire to develop a tool that can identify which regions can be stably reconstructed if the projection measurements are not complete. It is clear that the condition number is disqualified, because the condition number only tells whether the entire system can be stably solved as a whole. Even one pixel (i.e., one unknown) is unsolvable, the condition number is extremely large or infinity. If the condition number is infinity and not all pixels can be solved, we ask a further question: “Are there any pixels that can be stably solved?”

The Moore-Penrose pseudo inverse matrix method is SVD based and is a powerful tool to use when some singular values of the system matrix are zero. However, the SVD method requires that the entire system matrix be stored in the computer memory during computation. In reality, the system matrices are too large to store. The SVD methods are not practical.

This paper proposed a practical tool that maps out the stably solvable regions in the image. The basic idea of the tool is to reconstruct a large number of random images and compute their errors with respect to their associated true images. In other words, this is a Monte Carlo based method, The errors are location dependent. The regions that have large errors are not solvable. This idea is similar to machine learning. Here we use a large number of phantoms to ‘train’ the image solvability map. The map is imaging-geometry dependent. If the imaging geometry is altered, we need to ‘re-train’ a new map for the new geometry. We must point out that the image solvability map is also reconstruction algorithm dependent.

## Figures and Tables

**FIGURE 4.**
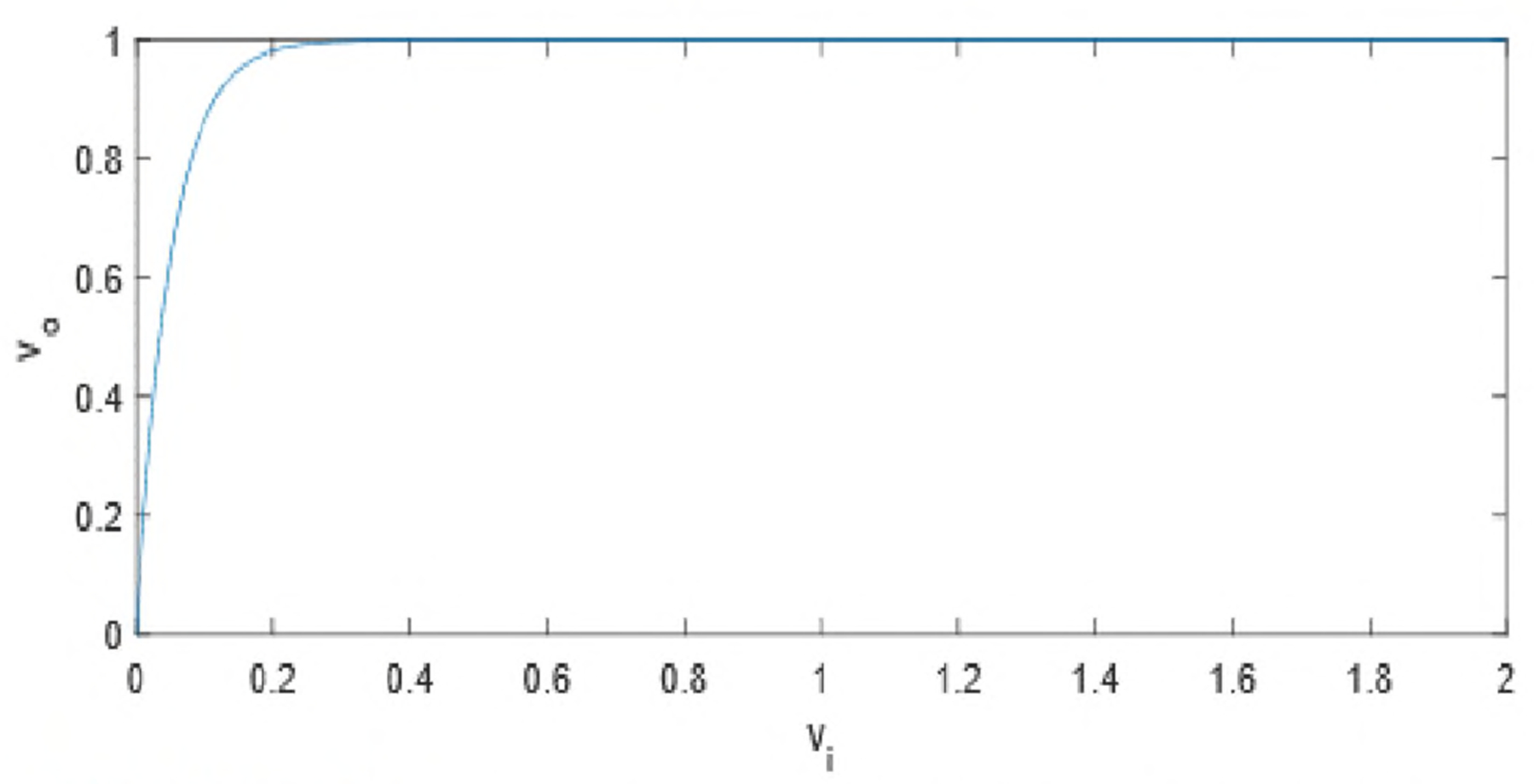
The image solvability map uses a nonlinear transformation to suppress the large values before the map is displayed.

**TABLE 1.** Maximum and minimum values in the image solvability map for the GD algorithms (see Fig. 10) using the truncated data

Algorithm	Minimum value	Maximum value
GD (1) with support (3) and truncation modification (5)	4.0035 × 10^−04^	0.0307
GD (1) with support (3)	0.0022	20.7523
GD (1) with truncation modification (5)	0.0022	0.0537
GD (1)	0.0034	20.9302

**TABLE 2.** Maximum and minimum values in the image solvability map for the ML-EM algorithms (see Fig. 11) using the truncated data

Algorithm	Minimum value	Maximum value
ML-EM (2) with support (4) and truncation modification (5)	4.1328 × 10^−04^	0.1168
ML-EM (2) with support (4)	6.3099 × 10^−04^	50.4166
ML-EM (2) with truncation modification (5)	3.5910 × 10^−04^	0.1734
ML-EM (2)	6.3049 × 10^−04^	50.4502

**TABLE 3.** Maximum and minimum values in the image solvability map for the GD algorithm (see Fig. 12) and the ML-EM algorithm (see Fig. 13) using the untruncated data

Algorithm	Minimum value	Maximum value
GD (1)	0.0020	0.0156
ML-EM (2)	1.8218 x 10^−04^	0.0249
